# 2-PMAP Ameliorates Cerebral Vasospasm and Brain Injury after Subarachnoid Hemorrhage by Regulating Neuro-Inflammation in Rats

**DOI:** 10.3390/cells11020242

**Published:** 2022-01-12

**Authors:** Chieh-Hsin Wu, Hung-Pei Tsai, Yu-Feng Su, Cheng-Yu Tsai, Ying-Yi Lu, Chih-Lung Lin

**Affiliations:** 1 Division of Neurosurgery, Department of Surgery, Kaohsiung Medical University Hospital, Kaohsiung 80756, Taiwan; 940065@kmuh.org.tw (C.-H.W.); 1060665@kmuh.org.tw (H.-P.T.); suyufeng@kmu.edu.tw (Y.-F.S.); 1030459@kmuh.org.tw (C.-Y.T.); 2 Department of Surgery, School of Medicine, College of Medicine, Kaohsiung Medical University, Kaohsiung 80756, Taiwan; 3 Department of Dermatology, Kaohsiung Veterans General Hospital, Kaohsiung 813, Taiwan; 4 Department of Health and Beauty, Shu-Zen Junior College of Medicine and Management, Kaohsiung 821, Taiwan

**Keywords:** 2-PMAP, apoptosis, brain injury, neuro-inflammation, subarachnoid hemorrhage (SAH), vasospasm

## Abstract

A subarachnoid hemorrhage (SAH), leading to severe disability and high fatality in survivors, is a devastating disease. Neuro-inflammation, a critical mechanism of cerebral vasospasm and brain injury from SAH, is tightly related to prognoses. Interestingly, studies indicate that 2-[(pyridine-2-ylmethyl)-amino]-phenol (2-PMAP) crosses the blood–brain barrier easily. Here, we investigated whether the vasodilatory and neuroprotective roles of 2-PMAP were observed in SAH rats. Rats were assigned to three groups: sham, SAH and SAH+2-PMAP. SAHs were induced by a cisterna magna injection. In the SAH+2-PMAP group, 5 mg/kg 2-PMAP was injected into the subarachnoid space before SAH induction. The administration of 2-PMAP markedly ameliorated cerebral vasospasm and decreased endothelial apoptosis 48 h after SAH. Meanwhile, 2-PMAP decreased the severity of neurological impairments and neuronal apoptosis after SAH. Furthermore, 2-PMAP decreased the activation of microglia and astrocytes, expressions of TLR-4 and p-NF-κB, inflammatory markers (TNF-α, IL-1β and IL-6) and reactive oxygen species. This study is the first to confirm that 2-PMAP has vasodilatory and neuroprotective effects in a rat model of SAH. Taken together, the experimental results indicate that 2-PMAP treatment attenuates neuro-inflammation, oxidative stress and cerebral vasospasm, in addition to ameliorating neurological deficits, and that these attenuating and ameliorating effects are conferred through the TLR-4/NF-κB pathway.

## 1. Introduction

Aneurysmal subarachnoid hemorrhage (SAH) is a deadly cerebrovascular disease [1] with a mean age of 35 years, the incidence of which increases 1.06-fold with every year of age [2]. In elderly patients the risk of fatality and adverse outcomes after SAH increase by 6% and 11%, respectively, with age [3]. After surviving a first SAH, cerebral vasospasm and early brain injury (EBI) contribute to subsequent morbidity and death for the most part [4,5,6]. Only two-thirds of survivors are functionally independent 1 year after SAH [7]. Notably, one study has demonstrated that, after SAH, apoptosis participates in aneurysm formation, vasospasm and EBI [8]. After global ischemia, patients with SAH exhibit apoptosis in the vasculature, blood–brain barrier (BBB) and brain [9]. Mechanisms of secondary brain injury after SAH are multifactorial.

After SAH, neuro-inflammation is an important pathological mechanism in vasospasm and brain injury [10]. Global hypoperfusion initially induces inflammatory processes in both blood vessels and cerebrospinal fluid (CSF) [11]. Inflammatory responses characterized by the release of pro-inflammatory mediators and the activation of resident microglia and astrocytes exacerbate BBB disruption. These responses further worsen the inflammatory reactions and neurological impairments [12]. The breakdown of red blood cells activates Toll-like receptor-4 (TLR-4) [13], which induces inflammatory responses resulting from interaction with nuclear factor kappa-light-chain-enhancer of activated B cells (NF-kB), which signals inflammatory cascades that damage nerve tissues [14,15]. NF-kB is the transcription factor known to have the largest regulatory role in the induction of inflammation-related genes in intracranial aneurysm lesions [16]. Microglia in the central nervous system are resident macrophages with various functions [17]. The M1 microglia induce inflammation, while M2 microglia restore brain tissue homeostasis [18]. NF-κB signaling contributes to the polarization of microglia and the secretion of pro-inflammatory cytokines [19]. Reducing neuronal apoptosis and inhibiting inflammation can potentially improve neurological recovery after SAH [20]. Therefore, enhancing protection against neuro-inflammation may be an effective strategy for treating SAH.

Treatment with 2-[(pyridine-2-ylmethyl)-amino]-phenol (2-PMAP) is reportedly a possible alternative therapeutic treatment for Alzheimer’s disease (AD) mice models [21]. The neuropathology of AD manifests as the progressive deposition of β-amyloid (Aβ) peptides in the brain parenchyma. Hence, neurofibrillary tangles evolve with the loss of synapses, paralleling local inflammatory responses [22]. Interestingly, 2-PMAP can inhibit amyloid precursor protein (APP) synthesis and Aβ secretion in CHO APP751SW cells. Since 2-PMAP apparently has no toxic effects and penetrates the BBB, 2-PMAP administered in a transgenic mouse model of AD can also reduce full-length APP and soluble Aβ accumulation in the brain. A novel finding is that 2-PMAP can improve mouse objective and spatial memory in a radial arm maze, which suggests that 2-PMAP may directly regulate brain function. 

This study established an SAH rat model to explore whether 2-PMAP ameliorates vasospasm and brain injury after SAH and whether it improves neurological functioning after SAH. The experiments proved that 2-PMAP has potential use for reducing apoptosis and attenuating neuroinflammation in the brain. Therefore, 2-PMAP has potential use not only for treating vasospasm induced by SAH, but also for treating ischemia-induced brain injury after SAH.

## 2. Materials and Methods

### 2.1. Ethics Statement

All procedures in experimental animals complied with the relevant ethical protocols approved by the Institutional Animal Care and Use Committee of Kaohsiung Medical University. All experimental protocols also complied with ARRIVE guidelines.

### 2.2. Animal Preparation

2-PMAP (Sigma; 102212-26-0; St. Louis, MO, USA) was resynthesized from 2-aminophenol and 2-pyridine carboxaldehyde, and then purified with a commercially available flash column chromatograph. All animal studies that were conducted utilized 2-PMAP with ≥ 98% purity, confirmed by HPLC/UV analysis. Male Sprague Dawley rats weighing 350 to 450 g (BioLasco, Taiwan Co., Ltd., Taipei, Taiwan) were randomly assigned into three groups (six rats per group): (1) a group with sham treatment (non-SAH group); (2) a group with SAH treatment (SAH group); and (3) a group with SAH treatment and 2-PMAP treatment (SAH+2-PMAP group). The SAH+2-PMAP group was pretreated with 5 mg/kg 2-PMAP injected into the subarachnoid space before the induction of SAH. The non-SAH group served as a control. The animals were housed at a constant temperature (24 °C) in a 12 h light/dark cycle, with free access to food and water.

### 2.3. SAH Model

Experiments were performed using a single-injection cisterna magna rat model. By an intraperitoneal injection of Zoletil 50^®^ (40 mg/kg, Virbac, Carros cedex), rats were anesthetized. The rats were then put in a prone head-down position, and a 25-gauge butterfly needle was inserted into the cisterna magna to slowly aspirate the cerebrospinal fluid (0.3 mL). Through the butterfly needle, the autologous fresh non-heparinized blood (0.1 mL/100 g of body weight), withdrawn from the central tail artery, was slowly injected into the subarachnoid space. The rats were then placed in the ventral recumbent position for 30 min to ensure ventral blood distribution. Then, they were kept warm with maintenance of the body temperature at 36 ± 1 °C until they recovered. The sham group received the same surgical procedure without blood injection. Two days after SAH, samples were collected for analysis.

### 2.4. Neurological Scores

Neurological function was evaluated 48 h after SAH by motor function (Table 1), which scored ambulation from 0 to 4 and placing/stepping response from 0 to 2 [23]. By walking with lower extremities, ambulation was assessed; by dragging the hind paw dorsal surface edge, placing/stepping reflex was also evaluated [24]. Total scores of motor deficits index (MDI) was determined by two blinded investigators.

### 2.5. Tissue Processing

Each rat was anesthetized again at the end of experiments for perfusion and fixation; the chest was opened and a 16 G catheter was inserted into the left ventricle. Next, the rat was perfused with phosphate-buffered saline (PBS) and fixed with 4% paraformaldehyde for BA morphometric studies and TUNEL staining. The brain tissues were removed and postfixed in 4% paraformaldehyde with 30% sucrose overnight. At last, these tissues were dehydrated and embedded in a Tissue-Tek^®^ optimal cutting temperature compound. Furthermore, the rat was perfused with phosphate-buffered saline (PBS) for Western blotting analysis. The brain tissues were removed and further washed in ice-cool PBS. Basilar artery tissues were removed and the whole brain tissues were cut into slices. The degree of cerebral vasospasm was blindly measured by physicians using measurements of the cross-sectional area and intima-media thickness of the basilar artery, calculating their relative ratio.

### 2.6. BA Morphometric Studies

The middle-third part of the BA was dissected for analyzing the extent of vasospasm. The BA cross-sectional area and intima-media thickness were scanned and analyzed by computerized image analysis. The BA cross-sectional area was measured by tracing the entire internal arterial lumen, and at least six random areas were qualitatively assessed and averaged in each rat. The intima-media thickness was measured as the maximal distance between the internal intima and the outer adventitia. The ratio of the cross-sectional area to the intima-media thickness was analyzed to assess the severity of vasospasm based on previous studies from the literature [9,25,26,27]. 

### 2.7. Immunofluorescence Staining

To retrieve antigens with a DAKO antigen retrieval solution (DAKO, Carpenteria, CA, USA), brain samples were heated by steam for 30 min. To inhibit endogenous peroxidase, a series of slices were washed with Tris-buffered saline (TBS) and immersed in a 3% hydrogen peroxide solution for 10 min. Slices were then incubated with a TUNEL kit and primary antibodies (mouse anti-NeuN antibodies (Merck; MAB377; Darmstadt, Germany); rabbit anti-Iba1 (proteintech; 10904-1-AP; Rosemont, IL, USA); and mouse anti-GFAP antibodies (Sigma; G3893; USA)) at room temperature. After being washed twice with TBS, the slices were incubated with anti-mouse antibodies (Thermo; A10524; Waltham, MA, USA) and anti-rabbit antibodies (Thermo; A11008; Waltham, MA, USA) at room temperature for 3 h. Next, the slices were stained and mounted within Fluroshield TM with DAPI (Sigma; F6057; USA) after being washed twice with TBS. Images were captured with a fluorescence microscope (Olympus, U-RFL-T) and the fluorescence intensity was measured by Image J vers. 1.44d software (NIH).

### 2.8. Western Blotting Analysis

The protein of brain tissue was extracted from each rat using an ice-cold lysis buffer containing protease inhibitors (Sigma; St. Louis, MO, USA), and the concentrations in the supernatant were determined with a protein assay kit (Bio-Rad Laboratories). Next, an equal amount of total protein was put into each well, separated with a 10% SDS-PAGE and further transferred onto a polyvinylidene fluoride membrane. After that, the membrane was blocked for 1 h with TBST and 5% skim milk at room temperature, and was further incubated at 4 °C overnight with primary antibodies (TLR-4 (1:500; 19811-1-AP; Proteintech, Rosemont, IL, USA), NF-κB (1:500; 10745-1-AP; Proteintech), p-NF-κB (1:500; #3033; Cell signalling), cleaved caspase-3 (1:400; #9661; Cell Signaling), Bcl-2(1:500; #3498; Cell Signaling), Bax (1:2000; 60267-1-Ig; Proteintech,) and β-actin (1 : 20000; A5441; Sigma)). Then, corresponding secondary antibodies (goat anti-rabbit IgG (1:2000; 111-035-444; Jackson ImmunoResearch, West Grove, PA, USA) and goat anti-mouse IgG (1:2000; AP124P; Jackson ImmunoResearch, West Grove, PA, USA)) were probed for 1 h at 25 °C after the membrane was rinsed for several times. Ultimately, the MiniChemi™ chemiluminescent system (Sage Creation Science, Beijing, China) was applied to measure the band density.

### 2.9. Quantitative Real-Time Polymerase Chain Reaction (qRT-PCR)

Total RNA was extracted from the BA and brain tissues with a TOOLSmart RNA Extractor (Biotools, Taiwan), and a TOOLS Easy Fast RT Kit (Biotools, New Taipei City, Taiwan) was then employed to reverse-transcribe the total RNA to cDNA. The cDNA was amplified with primers manufactured by Sigma (Darmstadt, Germany). GAPDH (glyceraldehydes-3-phosphate dehydrogenase) was used as the internal reference gene. The target genes and GAPDH were amplified by PCR performed with TOOLS 2X SYBR qPCR Mix. The primer sequences were as follows: TNF-α: forward: 5′-GCCCAGACCCTCACACTC-3′ and reverse: 5′-CACTCCAGCTGCTCCTCT-3′; IL-1β: forward: 5′-CACCTTCTTTTCCTTCATCTTTG-3′ and reverse: 5 ′-GTCGTTGCTTGT CTCTCCTTGTA-3′; IL-6: forward: 5′-CCGGAGAGGAGACTTCACAG-3′ and reverse: 5′-ACAGTGCATCATCGCTGTTC-3′; TLR-4: forward: 5′-TGCTCAGACATGGCAGTTTC- 3′ and reverse sequence: 5′-TCAAGGCTTTTCCATCCAAC -3′; GAPDH: forward: 5′-AGACAGCCGCATCTTCTTGT-3′ and reverse: 5′-CTTGCCGTGGGTAGAGTCAT-3′. The RT-PCR was performed on a Step One Plus Real-Time PCR system using Step One software, V2.3 (Applied Biosystems, Waltham, MA, USA).

### 2.10. Enzyme-Linked Immunosorbent Assay (ELISA)

Forty-eight hours after SAH, CSF samples were collected and centrifuged immediately at 2000× *g* at 4 °C for 10 min to remove cells, then stored below −15 °C until analysis. After passing samples through C2 columns (Amersham, UK; Nutley, NJ, USA), cytokine levels (TNF-α, IL-1β and IL-6) were determined at 450 nm by ELISA kits (Amersham, UK).

### 2.11. Measurement of Reactive Oxygen Species (ROS)

A Superoxide Detection Assay Kit (ab139476; abcam, Cambridge, MA, USA) was applied to measure ROS in brain tissues. Slices of brain tissues were incubated with 1 mL of 5 µM oxidative stress detection reagent at 37 °C for 10 min protected from light. After staining, the slices were observed under a microscope (Olympus, Tokyo, Japan) to determine the amount of ROS generated from mitochondria.

### 2.12. Statistical Analyses

All data were processed using SPSS software and presented as group means ± standard deviation (SD). Comparisons between groups were performed by a one-way ANOVA or Mann–Whitney U test for data with or without a normal distribution. Differences were considered significant for *p*-value less than 0.05.

## 3. Results

### 3.1. Mortality and Neurological Deficit in SAH Rats

A total of 36 rats were used for these studies. After SAH, thick blood clots were seen over the basal surface of the brain stem. Two days after SAH, no rats died. To determine how 2-PMAP affected brain injury in the SAH rats, the MDI was determined to evaluate the neurological deficits. According to the examination results, both scores in the SAH groups were significantly higher than that of the sham group. The values of the MDI in the SAH were 4.33 ± 1, compared with a score of 0 in the sham group. Treatment with 2-PMAP significantly improved the MDI (Table 2). Therefore, the results indicated that 2-PMAP treatment protected against the impairment of neurological function in SAH rats.

### 3.2. 2-PMAP Reduced Vasospasm Severity in SAH Rats

**Figure 1:**
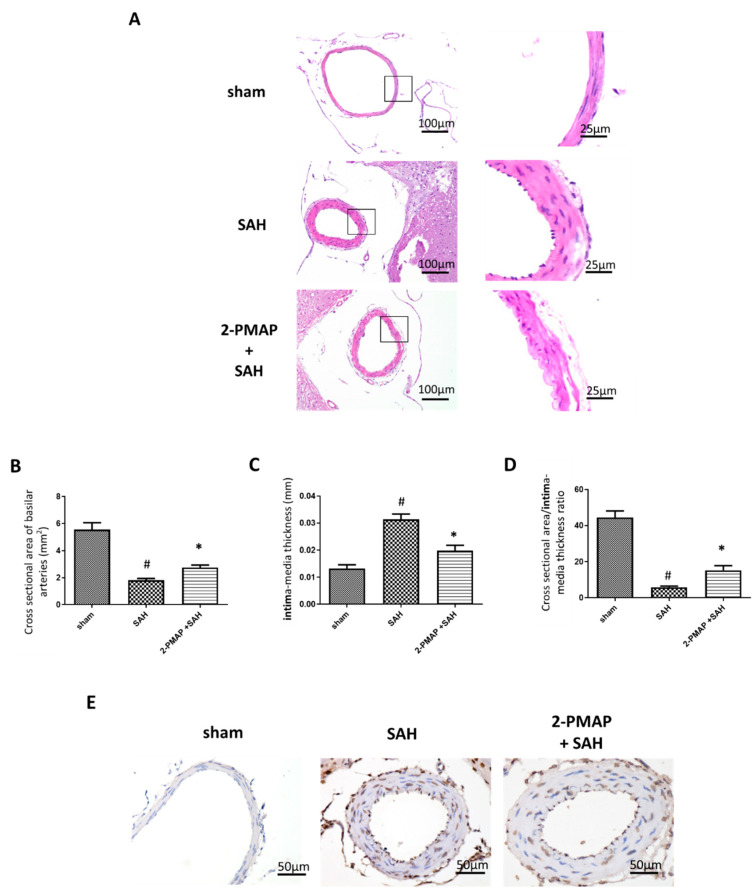
2-PMAP reduced vasospasm severity in SAH rats. (**A**) Representative cross-sections of the BA in SAH rats by H&E stain. Left panels have scale bars of 100 μm. Right panels are high-power views of tissues, with scale bars of 25 μm. (**B**–**D**) Quantification of the (**B**) cross-sectional areas of the internal lumen, (**C**) the BA wall intima-media thickness and (**D**) the ratio of the cross-sectional area to the wall intima-media thickness. Data are shown as mean + SD (*n* = 6 for each group); *, *p* < 0.05, significantly different from the SAH group. #, *p* < 0.05, significantly different from the sham group. (**E**) TUNEL staining was utilized to detect apoptotic endothelial cells. Scale bars: 50 μm. No apoptotic endothelial cells of the BA were observed in sham rats. Increased apoptotic endothelial cells were observed in SAH rats, which was reduced markedly by 2-PMAP (arrows).

Cerebral vasospasm in SAH rats was evaluated by measuring the cross-sectional areas of the BA in individual rats 48 h after blood injection into the cisterna magna (Figure 1A). In the sham group, the tunica media of the BA had an orderly arrangement of smooth muscle cells. In the SAH group, the BA revealed marked curling and thickening in the elastic lamina and marked swelling in endothelial cells. In contrast, the SAH+2-PMAP group only had mild curling of the elastic lamina and mild swelling of endothelial cells. Figure 1B compared the mean BA cross-sectional area in the three groups. The BA had a significantly smaller mean cross-sectional area in the SAH group compared to the sham group, but improved after treatment with 2-PMAP. Figure 1C showed that the vessel wall intima-media thickness was significantly lower in the SAH+2-PMAP group compared to the SAH group. Finally, vasospasm severity was estimated by calculating the ratio of the BA cross-sectional area to the BA wall intima-media thickness. Again, the SAH+2-PMAP group had a significantly decreased ratio compared to the SAH group (Figure 1D). Notably, the TUNEL staining results for BA endothelial cells in the sham group were negative. At 48 h after SAH, TUNEL-positive cells appeared in the BA in SAH rats. Treatment with 2-PMAP remarkably reduced these cells. By attenuating BA endothelial apoptosis, 2-PMAP was able to improve cerebral vasospasm after SAH. 

### 3.3. 2-PMAP Decreased Apoptotic Neurons in SAH Rats

**Figure 2:**
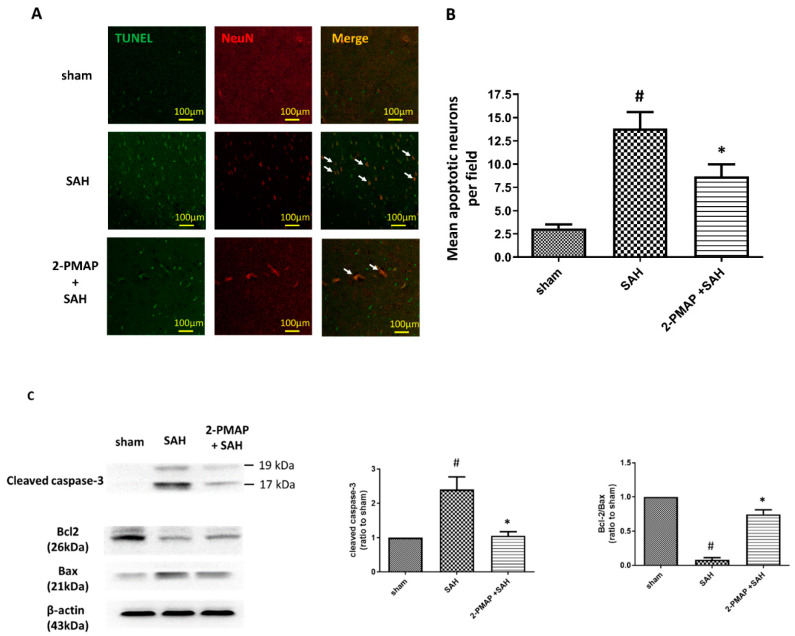
2-PMAP decreased apoptotic neurons in SAH rats. (**A**) TUNEL staining was utilized to detect apoptotic neurons in SAH rats after 2-PMAP treatment. Scale bars: 100 μm. NeuN was used as a neuron marker. Representative immunofluorescence staining of neuronal nuclei (NeuN, red) and cell death (TUNEL, green). Images of each group are displayed as single staining results for NeuN and TUNEL, and a fused image displays both cells (green and red). SAH induced the development of apoptotic neurons in the brain tissues, whereas 2-PMAP decreased neuronal apoptosis (arrows). (**B**) Percentage of apoptotic neurons in the brain tissues 48 h after SAH. (**C**) Western blotting was utilized to measure the protein levels of cleaved caspase-3, Bcl-2 and Bax in brain tissues. Left panel: representative expression of cleaved caspase-3, Bcl-2 and Bax protein of brain tissues. Beta-actin was used as its internal control. Right panel: quantification of cleaved caspase-3 and relative ratio of Bcl-2 to Bax activity. Data are shown as mean ± SD (*n* = 6 for each group); *, *p* < 0.05, significantly different from the SAH group. #, *p* < 0.05, significantly different from sham group.

An SAH can cause apoptosis in the cerebral cortex, with poor prognoses [9,28]. In this work, the protective effect of 2PMAP against neuronal apoptosis was evaluated by performing TUNEL staining 48 h after SAH with neurons marked by NeuN. No apoptosis occurred in the sham group (Figure 2A), whereas the SAH group had significantly higher apoptotic neuron counts. Figure 2B shows that neuronal apoptosis was substantially lower in the SAH+2-PMAP group compared to the SAH group.

On the other hand, the protein expression of cleaved caspase-3, Bcl-2 and Bax in brain tissues were measured by Western blotting. SAH induced significant increases in cleaved caspase-3 in the brain tissues of the rats (approximately 2.5-fold higher compared to the sham group) and decreased the ratio of Bcl-2 to Bax; compared to the SAH group, the administration of 2-PMAP markedly reversed these expressions (Figure 2C). 

Taken together, these results suggested that 2-PMAP administration substantially reduced the neuronal damage caused by SAH in the experimental rats.

### 3.4. 2-PMAP Decreased Inflammatory Cytokines in SAH Rats

**Figure 3:**
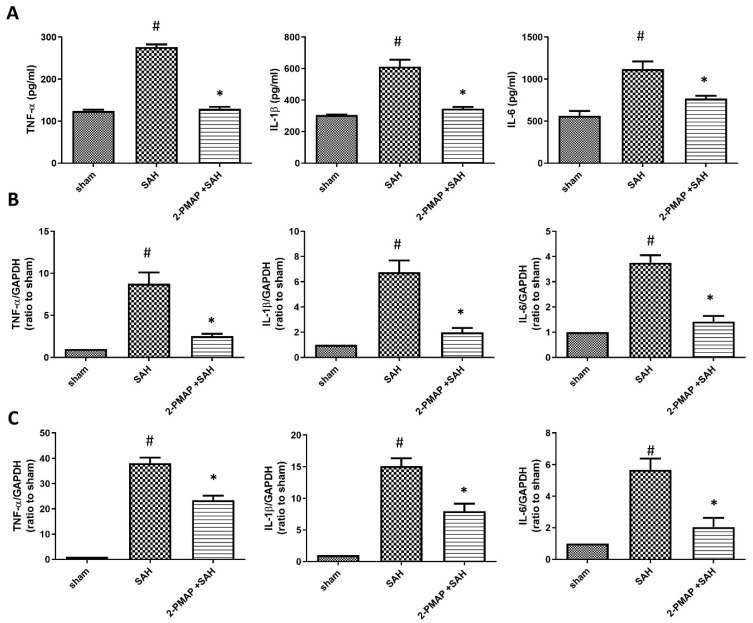
2-PMAP decreased inflammatory cytokines in SAH rats. (**A**) The inflammatory cytokines in CSF were measured by an enzyme-linked immunosorbent assay 48 h after SAH. The level of TNFα, IL-1β and IL-6 was significantly increased after SAH, whereas 2-PMAP reversed these levels. (**B**,**C**) qRT-PCR was used to test the relative expression of inflammatory cytokines in the BA and brain tissues. The level of *TNFα, IL-1β* and *IL-6* in (**B**) the BA and (**C**) brain tissues was significantly increased after SAH, whereas 2-PMAP also reduced these levels. Data are shown as mean ± SD (*n* = 6 for each group); *, *p* < 0.05, significantly different from the SAH group. #, *p* < 0.05, significantly different from the sham group.

Next, we performed an ELISA to characterize inflammatory cytokines in CSF, which is implicated in the pathology of SAH (Figure 3A). At 2 days after the induction of SAH, the SAH group had significantly higher levels of pro-inflammatory cytokines compared to the sham group (TNFα and IL-1β were approximately three-fold higher compared to the sham group and IL-6 was approximately two-fold higher compared to sham group). However, the expression of these cytokines was significantly lower in the SAH+2-PMAP group compared to the SAH group. 

Similarly, we performed qRT-PCR to analyze the level of inflammatory cytokines in the brain tissues and BA, which also contribute to the pathology of SAH. At 2 days after the induction of SAH, the SAH group had significantly higher levels of pro-inflammatory cytokines compared to the sham group in brain tissues (*TNFα, IL-1β* and *IL-6* were approximately 10-fold, 7-fold and 4-fold higher compared to the sham group, respectively) (Figure 3B) and the BA (*TNFα, IL-1β* and *IL-6* were approximately 35-fold, 15-fold and 6-fold higher compared to the sham group, respectively) (Figure 3C). However, the expression of these cytokines was also significantly lower in the SAH+2-PMAP group compared to the SAH group in brain tissues and the BA. 

**Figure 4:**
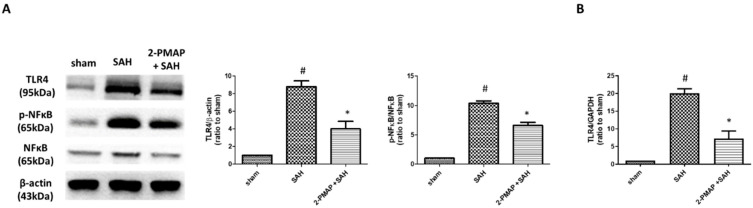
2-PMAP reduced the TLR-4/NF-κB signals in SAH rats. (**A**) Western blotting was utilized to measure the protein levels of TLR-4, NF-κB and phospho-NF-κB in brain tissues. Left panel: representative expression of TLR-4, NF-κB and phospho-NF-κB protein of brain tissues. Beta-actin was used as an internal control. Right panel: quantification of TLR-4 and relative p-NK-κB activity. (**B**) qRT-PCR was used to test the relative expression of *TLR-4* in the BA. The level of *TLR-4* in the BA was significantly increased after SAH, whereas 2-PMAP also reduced the level. Data are shown as mean ± SD (*n* = 6 for each group); *, *p* < 0.05, significantly different from the SAH group. #, *p* < 0.05, significantly different from the sham group.

On the other hand, the protein expression of TLR-4 and p-NF-κB/NF-κB in brain tissues were measured by Western blotting. SAH induced significant increases in the brain tissues of the rats (TLR-4 and p-NF-κB/NF-κB were approximately 10-fold higher compared to the sham group); compared to the SAH group, the administration of 2-PMAP markedly reduced these expressions (Figure 4A). Besides, the expression of *TLR-4* in the BA was measured by qRT-PCR. SAH induced significant increases in the BA of the rats (*TLR-4* was approximately 20-fold higher compared to the sham group); compared to the SAH group, the administration of 2-PMAP markedly reduced the expression (Figure 4B). Taken together, by ameliorating apoptosis through regulating inflammatory cytokines, 2-PMAP improved neurobehavioral outcomes after SAH.

### 3.5. 2-PMAP Inhibited Microglia Activation in SAH Rats

**Figure 5:**
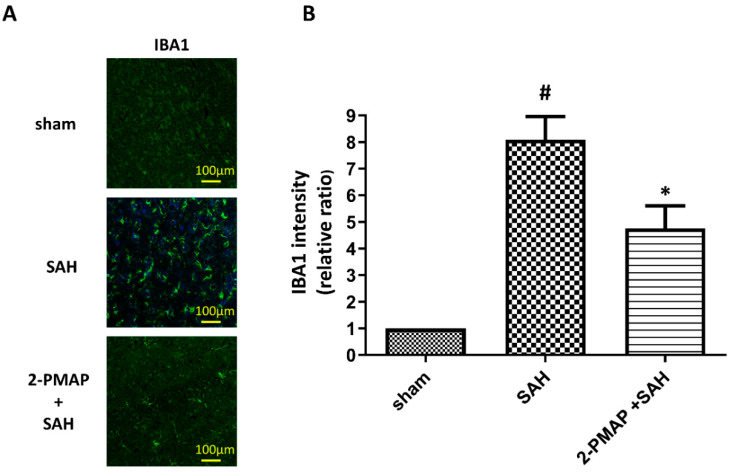
2-PMAP inhibited microglia activation in SAH rats. Slices of brain tissues were prepared in rats treated with the administration of 2-PMAP, or not, 48 h after the SAH. Immunofluorescence staining showing microglia activation in brain tissues after SAH. (**A**) Representative images for activated microglia in the brain tissues, which show ameboid morphology of IBA1-positive microglia. Scale bars: 100 μm. (**B**) Relative fluorescence intensity of IBA-1. Data are shown as mean ± SD (*n* = 6 for each group); *, *p* < 0.05, significantly different from the SAH group. #, *p* < 0.05, significantly different from the sham group.

Since SAH may induce microglia activation within brain parenchyma, the inflammation status of brain tissues was measured by immunofluorescence staining. Figure 5 shows that there were relatively low IBA1-positive microglia existing in the sham group. As compared with the sham group, a significantly greater number of IBA1-positive microglia appeared in the SAH group with amoeboid morphology and activated microglia, whereas 2-PMAP recovered the number of activated microglia. These results suggest microglia activated in the brain after SAH and was markedly reversed after 2-PMAP treatment.

### 3.6. 2-PMAP Decreased Astrocyte Activation in SAH Rats

**Figure 6:**
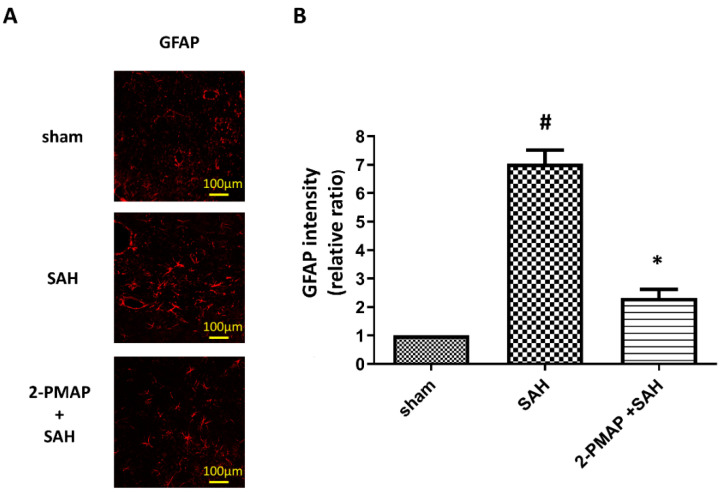
2-PMAP decreased astrocyte activation in SAH rats. Slices of brain tissues were prepared in rats treated with the administration of 2-PMAP, or not, 48 h after the SAH. Immunofluorescence staining showing reactive astrogliosis in brain tissues after SAH. (**A**) Representative images of reactive astrogliosis in the brain tissues. Scale bars: 100 µm. (**B**) Relative fluorescence intensity of GFAP. Data are shown as mean ± SD (*n* = 6 for each group); *, *p* < 0.05, significantly different from the SAH group. #, *p* < 0.05, significantly different from the sham group.

Reactive astrogliosis (upregulation of glial fibrillary acidic protein (GFAP)) is a consequence of microglia activation, enhancing the inflammatory response in SAH [29]. Figure 6 shows SAH significantly induced a nearly seven-fold increase in GFAP expression compared to the sham group, whereas 2-PMAP reversed the number of reactive astrocytes. These results suggest reactive astrocytosis was induced in the brain after SAH, and was markedly improved after 2-PMAP treatment.

### 3.7. 2-PMAP Decreased ROS in SAH Rats

**Figure 7:**
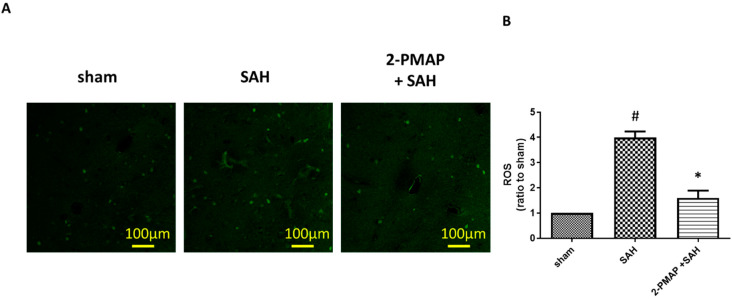
2-PMAP decreased reactive oxygen synthase (ROS) in SAH rats. Slices of brain tissues were prepared in rats treated with the administration of 2-PMAP, or not, 48 h after the SAH. Immunofluorescence staining showing ROS elevated in brain tissues after SAH. (**A**) Representative images of ROS in the brain tissues. Scale bars: 100 μm. (**B**) Relative fluorescence intensity of ROS. Data are shown as mean ± SD (*n* = 6 for each group). *, *p* < 0.05, significantly different from the SAH group. #, *p* < 0.05, significantly different from the sham group.

Mitochondrial dysfunction leads to the overproduction oxygen radicals, the release of apoptogenic proteins and the generation of mitochondria-related inflammation, which are associated with brain injury after SAH [30,31]. Figure 7 shows SAH significantly induced a nearly four-fold increase in the production of ROS as compared to the sham group, whereas 2-PMAP reduced these elevated levels. These results suggest mitochondrial dysfunction was induced in the brain after SAH and was markedly improved after 2-PMAP treatment, which is consistent with the brain neuronal apoptosis. 

## 4. Discussion

**Figure 8:**
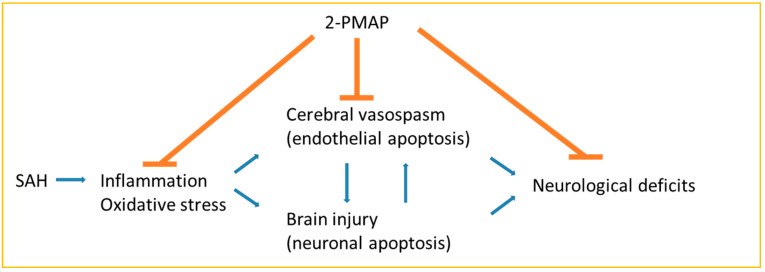
Schematic diagram of the regulatory role of 2-PMAP in SAH rats.

In the present study, treatment with 2-PMAP significantly suppressed vasospasm through decreasing apoptotic endothelial cells 2 days after SAH in rats. Meanwhile, 2-PMAP ameliorated microglia and astrocyte activation as well as neuro-inflammation (p-NF-κB/NF-κB, TLR-4, TNF-α, IL-1β and IL-6)) and oxidative stress, leading to a decrease in neuronal apoptosis in the brain cortex, which demonstrated that 2-PMAP was a promising drug to attenuate vasospasm and brain injury in SAH rats. This is the first study to explore the vasodilatory effect and neuroprotective role of 2-PMAP after SAH (Figure 8).

Approximately 10 out of 100,000 people each year experience SAH with residual sequela and economic burdens [32]. Despite advanced surgical techniques, EBI and vasospasm remain the major causes of mortality and morbidity [9,33]. Vasospasm induces ischemic damage to brain, in which the outcomes depend on the extent of the damage [1]. Therapies designed to alleviate brain injury can be expected to reduce death and disability in SAH patients. The mortality rate and neurological deficits are important to evaluate the outcome after SAH. In our study, the mortality rates in SAH patients with and without 2-PMAP treatment were similar. However, neurological deficits only improved in SAH patients with 2-PMAP treatment. Microglial and astrocyte activation in addition to neuro-inflammation are also consistent with the occurrence of vasospasm or neurobehavioral deficits [18].

After SAH, two major changes in vascular wall structure, endothelial apoptosis and smooth muscle cell proliferation, promote vascular remodeling in spastic vessels [34,35,36]. Endothelial apoptosis in major cerebral arteries initiates and maintains cerebral vasospasm [37,38,39,40]. It further induces anti-apoptotic factors to release and act on smooth muscle cells [41]. Hence, smooth muscle cells proliferate, arterial walls thicken and vascular stiffening develops to enhance cerebral vasospasm [35]. In the rat model performed by Leclerc et al. (2018), vasospasm severity was highest at 48 h after a single cisterna magna injection [42]. Therefore, we defined vasospasm severity as measured at 2 days after SAH. In our study, the ratio of the BA cross-sectional area to the BA wall intima-media thickness was compared after 2-PMAP treatment in a rat model of SAH. 2-PMAP significantly increased the activity of vasodilators in vasospastic BAs 48 h after SAH. The TUNEL studies revealed that apoptotic endothelial cell counts differed between the SAH+2-PMAP group and the SAH group. This finding suggested that the anti-vasospastic effect of 2-PMAP was an apoptosis-dependent effect.

Apart from impaired cerebral perfusion, neuro-inflammation is considered the hallmark pathology of brain damage after SAH [43]. Free hemoglobin released in the subarachnoid space stimulates inflammatory responses, which then cause both cerebral vasospasm and oxidative burst [37,44,45,46]. TNF-α causes apoptosis of cerebral endothelial cells [47]. Endothelial cell injuries disrupt the BBB, which further accelerates the release of inflammatory cytokines (TNF-α, IL-1β and IL-6) into CSF. TNF-α contributes to the recruitment of inflammatory mediators, oxidative stress and cell death [48]. Elevated expressions of inflammatory cytokines in serum or in CSF are associated with poor SAH outcomes [49,50,51,52]. Of all the Toll-like receptors (TLRs) in SAH, TLR-4 is the most important, which is activated by heme, fibrinogen and heat shock proteins produced after SAH [53]. The abnormally high level of TLR-4 in peripheral blood mononuclear cells is related to a high incidence of cerebral vasospasm with delayed cerebral infarction, a high severity of SAH with poor functional recovery [54]. The TLR-4 interacts with MyD88 or toll-receptor-associated activator of interferon (TRIF) to activate inflammatory cytokine genes [55]. Through MyD88, TLR-4 induces early phase NF-κB activation, and the TRIF-dependent pathway induces late-phase NF-κB activation. The NF-κB signaling pathway regulates inflammation and BBB integrity in brain ischemia [56], in which the phosphorylated NF-κB is required to activate inflammatory responses [57,58]. In our study, p-NF-κB/NF-κB and TLR-4 protein levels were increased in brain tissues after SAH; 2-PMAP treatment decreased the expressions. Taken together, the results indicated that the TLR-4/NF-κB signals were involved in the anti-inflammatory effects in SAH of 2-PMAP. 

In patients with neuro-inflammation after SAH, activated microglia are the main source of cytokines in the CNS [59,60]. Microglia are resident macrophages or differentiated recruited monocytes in brain innate immunity. Their function can rapidly change according to physiological or pathophysiological needs [61,62,63]. Within 2 days after SAH, microglia are activated with monocyte recruitment. Whereas the activation of microglia prevents neuronal injury and promotes tissue repair, microglia hyperactivation also promotes cell death and neuronal dysfunction [64]. Hence, we hypothesize that monocytes activate and differentiate in response to SAH. Excessive microglia activation further causes the secretion of inflammatory factors, which aggravates brain injury. Therefore, preventing excess microglia activation can alleviate brain injury after SAH [18,65,66]. In our study, significant increases in microglia activation and inflammatory cytokines resulted in severe brain injury. Notably, 2-PMAP treatment impeded SAH-induced microglia activation and reduced expressions of TLR-4/p-NF-κB. These effects were also accompanied by reduced expressions of inflammatory factors (TNF-α, IL-1β and IL-6), which indicated the suppression of microglial activation and the inhibition of neuro-inflammation. Therefore, we concluded that 2-PMAP ameliorates brain injury after SAH by inhibiting microglia activation and NF-κB signaling, which then reduces the inflammatory response to SAH. 

Both human models [67] and animal models [44] of SAH indicate that neuronal apoptosis occurs after SAH. Besides apoptosis, oxidative stress contributes to brain injury after SAH [68]. Inside the cells, the electric chain of mitochondrial oxidative phosphorylation is the predominant source of ROS [69]. Excessive ROS production induces DNA damage, and the resulting oxidative stress environments further exacerbate the cell damages [70]. Mitochondrial dysfunction generates ROS, thereby breaking the mitochondrial membrane potential, which causes neuronal death [71]. In the intrinsic mitochondrial pathway, the activity of cleaved caspase 3 contributes to cell apoptosis to the greatest extent. Cell death signals result in not only mitochondrial dysfunction but also caspase pathways, which are downstream of the Bcl-2 family [72]. Since the pro-survival protein, Bcl-2, regulates the activation or cleavage of caspases, and further suppresses the formation of pro-apoptotic proteins, Bax and Bad, the ratio of Bcl-2 to Bax usually indicates whether cell survival or cell death is the most dominate event [9]. In our study, SAH not only induced the increased production of ROS and cleaved caspase-3 in the brain tissues of the rats but also decreased the ratio of Bcl-2 to Bax; the administration of 2-PMAP improved these expressions. These results suggest that upstream death signals were induced in the brain after SAH, further resulting in mitochondrial dysfunction and the activation of caspases; both were ameliorated after 2-PMAP treatment, which is consistent with the brain neuronal apoptosis and neurological deficits.

Although activated microglia and astrocytes have beneficial effects in the early stage after SAH, they also cause the activation of macrophages through the recruitment of monocytes and neutrophils. The entry of monocytes and neutrophils into the subarachnoid space after SAH activates and maintains early phase inflammation [10,18,66]. Astrocytes are essential to keep brain homeostasis by regulating the CNS immune system, by supporting neuronal development/survival and by modulating neurotransmission [73]. In the adult brain, astrocytes do not proliferate unless nerve tissue damage occurs [74]. In response to tissue injury, “reactive astrocytes” are activated to preserve tissue integrity. Reactive astrogliosis, characterized by alterations in phenotype and by increases in the size and number of astrocytes (upregulation of GFAP and S100B) [75], have both protective and deleterious influences on neurons [75,76]. They not only can decrease brain edema, preserve BBB and protect neurons from damage, but also ensue scar formation, enhance the release of cytokines and limit axonal regeneration. Astrocyte activation is a possible consequence of microglia activation, which can cause neuro-inflammation [31]. In SAH patients, GFAP and S100B are elevated in both CSF and serum [77,78], in which high S100B levels in CSF is associated with poor one-year clinical outcomes [79]. In our study, GFAP was significantly increased in SAH, but the increase was reversed by 2-PMAP treatment, which suggests that 2-PMAP can reduce astrocyte activation, which is consistent with microglial activation and brain injury. Therefore, 2-PMAP is likely to act against neuro-inflammation and brain injuries by blocking microglial and astrocyte activation. 

## 5. Conclusions

In summary, our findings in a rat model of SAH suggest that the administration of 2-PMAP may ameliorate inflammatory responses, cerebral vasospasm and improve neurological impairment after SAH. The neuroprotective effects of 2-PMAP were mediated by the TLR-4/NF-κB signaling pathway. This study is the first to demonstrate that exogenous 2-PMAP treatment may protect against neuro-inflammation in patients with SAH. 

## Figures and Tables

**Table 1:** Motor function examination.

Motor	Behavior	Score
Ambulation	Normal (symmetric and coordinated)	0
	Toes flat under the body while walking with ataxia	1
	Knuckle walking	2
	Movement in lower extremities but unable to knuckle walk	3
	No movement, dragging lower extremities	4
Placing/stepping reflex	Normal (coordinated lifting and placing response)	0
	Weak response	1
	No stepping	2

**Table 2:** Behavioral assessment.

Treatment	Ambulation	Placing/Stepping Reflex	MDI
Sham	0	0	0
SAH	2.56 ± 0.88 ^#^	1.78 ± 0.44 ^#^	4.33 ± 1 ^#^
2-PMAP + SAH	1.44 ± 0.53 *	1.22 ± 0.49 *	2.67 ± 0.71 *

Neurological function was evaluated 48 h after SAH by motor function, total scores of motor deficits index (ambulation and placing/stepping response). MDI, motor deficits index. Six rats per group. *, *p* < 0.05, significantly different from SAH group. ^#^, *p* < 0.05, significantly different from sham group.

## Data Availability

All data generated or analyzed during this study are included in this published article.
